# Effective Management of a Fractured Tooth in a Child Using an Aesthetic Pediatric Crown: A Case Report and Review of Literature

**DOI:** 10.1155/2024/6888443

**Published:** 2024-11-06

**Authors:** Stephan Lampl, Deepa Gurunathan, Deepak Mehta, Jogikalmat Krithikadatta, Desigar Moodley

**Affiliations:** ^1^Department of Pediatric and Preventive Dentistry, Saveetha Dental College, Saveetha Institute of Medical and Technical Sciennces, Chennai, India; ^2^Department of Conservative Dentistry, Saveetha Institute of Medical and Technical Sciences, Chennai, India; ^3^Department of Cariology, Saveetha Institute of Medical and Technical Sciences, Chennai, India; ^4^Scinmed Aesthetic and Education Centre, Cape Town, South Africa

**Keywords:** crown materials, dentistry, mixed dentition, pediatric crowns, pediatric dentistry, primary teeth, traumatic dental injuries

## Abstract

**Background:** Traumatic dental injuries in children present unique challenges, requiring evidence-based and individualized approaches. Accidental falls are a common cause, resulting in a spectrum of clinical presentations. Managing tooth injuries in mixed dentition adds complexity to treatment decisions.

**Methods:** This case report details the conservative management of an 8-year-old with a fractured permanent lateral incisor following a fall. The child's physical status was assessed, ruling out abuse and associated neurological issues. A novel pediatric full crown fabricated from a hybrid glass material was chosen for its minimally invasive preparation and aesthetic properties.

**Results:** Procedural details, including crown sizing, contouring, and bonding, are outlined. One-year follow-up revealed no discoloration, pain, or adverse effects. The hybrid glass material demonstrated longevity in bonding, maintaining both function and esthetics. The discussion encompasses the choice of crown materials and the advantages of hybrid glass material.

**Conclusion:** The case highlights successful conservative restoration in mixed dentition using a pediatric full crown with a hybrid glass material. This approach achieved functional and aesthetic improvements, preserved tooth vitality, and ensured patient satisfaction.

## 1. Introduction

Traumatic dental injuries in children, a major dental health problem across the globe, pose unique functional and psychosocial challenges [1, 2]. Treating this condition often requires the application of evidence-based diagnostic and treatment practices that are optimally contextualized and individualized to the specific requirements of a child. Predisposing factors of dental trauma could be related to the person's anatomic features such as increased overjet or inadequate lip coverage of the upper anterior teeth [3]. Accidental falls are common causes of these traumatic dental injuries in children [4, 5] and their heterogenous clinical presentations can be categorized into nine fracture and six luxation variants [6]. A conspicuous complexity in the management of traumatic dental injuries is that traumas to the primary and the permanent teeth are separate entities and hence treated separately [6]. Furthermore, the management of tooth injuries in children with mixed dentition can be challenging. The Dental Trauma Guide and Trauma Pathfinder provide useful recommendations for the diagnosis and treatment of these heterogeneous presentations [6]. Furthermore, recommendations from the American Academy of Pediatric Dentistry (AAPD) suggest the objective of treatment of primary and immature permanent teeth with large fractures is to maintain the pulp vitality for continued apexogenesis [6]. Along with this, preservation of the remaining tooth structure is an important restorative consideration in children with traumatic injury to and erupting teeth [7].

On the premise of available evidence that pediatric full crowns as compared to conventional restorations can conserve tooth structure and maintain pulp vitality, we treated an 8-year-old child with a fractured permanent lateral incisor using a pediatric full crown fabricated using a novel hybrid glass material. The outcomes seen in this child along with a review of literature on the crown materials for achieving optimal restoration of function and aesthetic outcomes, while preserving pulp vitality and remaining tooth structure are presented herein.

## 2. Case Report

**Figure 1:**
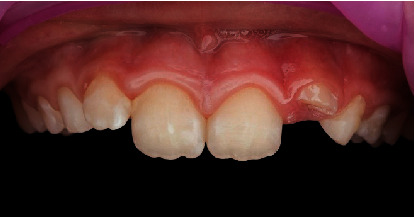
Preoperative clinical situation of fractured tooth (Number 22).

**Figure 2:**
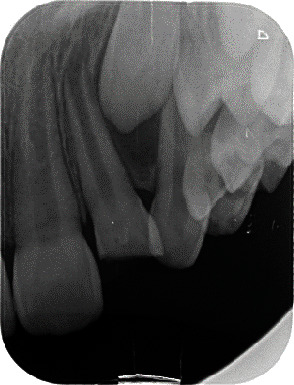
Periapical radiograph of the fractured tooth (Number 22).

Accompanied by her parents, an 8-year-old girl presented to our dental clinic with a fractured permanent lateral incisor in the left maxilla (Figures 1 and 2) caused by an accidental traumatic fall. After ruling out the possibility of child abuse, an assessment of physical status was made using the ASA Physical Status Classification System [8]. This child had no medical problems (ASA physical classification: 1), neurological decrements, or concussion-related consequences. Clinical examination of the face, lips, and oral musculature did not reveal any soft tissue lesions. Percussion tests indicated no tenderness, thus ruling out luxation or root fracture injury. Furthermore, evaluations of occlusion and bite force ruled out alveolar fractures and any other issues with tooth and jaw mobility. While pulp sensitivity was found to be normal, the child and her parents upon clinical elicitation confirmed the presence of pain and sensitivity to hot and cold drinks. This observation in the backdrop of normal pulp sensitivity and clinical examinations indicated the expected consequence of an exposed dentine. Furthermore, palpation of the jaws and clinical examinations revealed a traumatic fall-induced dentine fracture of the upper left lateral incisor (tooth Number 22), which was restricted to the enamel.

**Figure 3:**
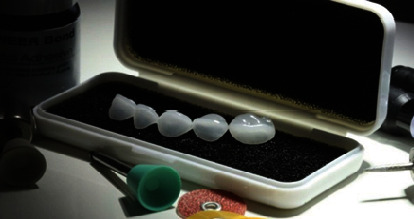
Edelweiss Paediatric Crowns as supplied by the manufacturer (used with permission from Edelweiss Dentistry).

In line with available evidence [7–9], a conservative treatment plan was arrived upon to treat the child's fractured tooth by preserving the remaining tooth structure. The overall treatment plan included steps for restoring function and esthetics while preserving tooth vitality. To implement this treatment plan, a novel pediatric full crown fabricated using a novel hybrid glass material was selected (Figure 3). This novel pediatric full crown requires minimal tooth preparation and conventional bonding agents for adhesion.

**Figure 4:**
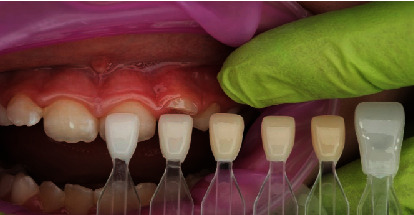
Edelweiss shade guide (used with permission from Edelweiss Dentistry).

**Figure 5:**
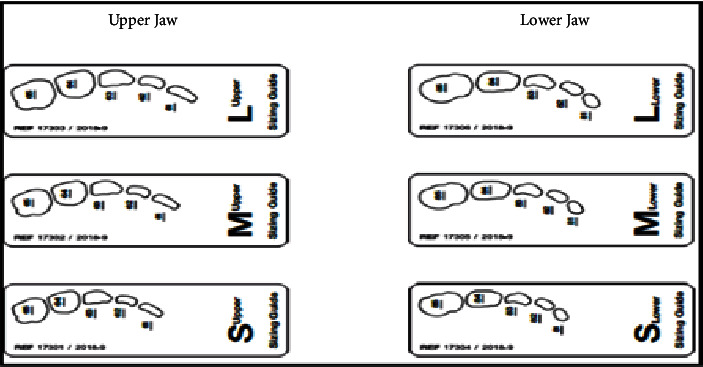
Edelweiss sizing guide (used with permission from Edelweiss Dentistry).

**Figure 6:**
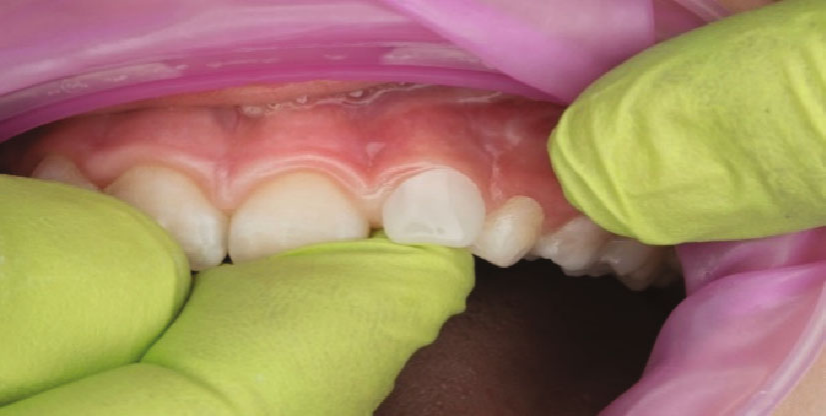
Edelweiss trial fit on tooth.

**Figure 7:**
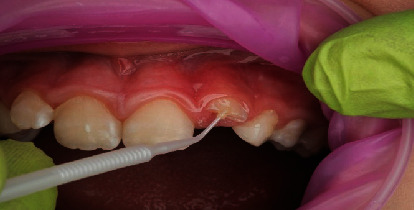
Application of dentine bonding agent to the etched surface.

**Figure 8:**
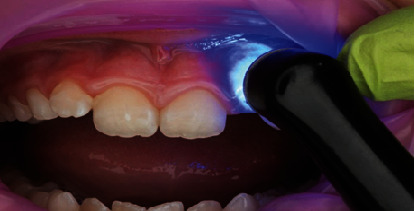
Light curing of bonding agent for 20 s.

In terms of procedural details, the manufacturer's shade and sizing guides (Figures 4 and 5) were utilized to determine an approximate crown size, and Shade A1 (see Figure 4) was found suitable. The margin of the pediatric full crown was then contoured to align with the existing gingival margin, employing a diamond football bur (RA 379, Komet, Lemgo, North Rhine-Westphalia, Germany) and Soflex discs (3M, St. Paul, MN, United States) on a slow handpiece. To facilitate proper oral care, the crown's margin was maintained in a supragingival position. Subsequently, the fitting surface of the pediatric crown was lightly roughened after confirming the gingival contour and the accuracy of fit on the fractured tooth (Figure 6). The roughening process involved using a diamond football bur (RA 379, Komet, Lemgo, North Rhine-Westphalia, Germany) to lightly remove the glossy inner layer. After rinsing and gently drying, a small amount of veneer bond (Edelweiss Dentistry Product, Graz, Austria) was applied to the roughened surface, followed by light curing for 20 s using a Valo Light Curing Unit (Ultradent, South Jordan, UT, United States). On the tooth side, the enamel surface was etched with 37% phosphoric acid (Ultra-etch, Ultradent, South Jordan, UT, United States) for 10 s, thoroughly rinsed, and gently air-dried. A universal adhesive bonding (All Bond Universal Adhesive, Bisco, Schaumburg, IL, United States) was applied to the enamel and dentine surfaces, and the surface was light cured for 20 s in all directions (Figures 7 and 8).

**Figure 9:**
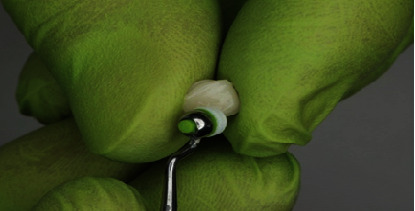
Edelweiss composite applied to the inside of the pediatric crown.

**Figure 10:**
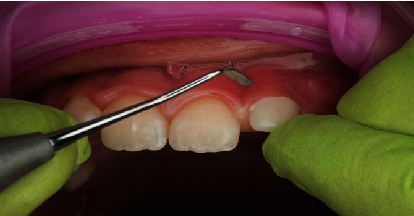
Excess composite removed from the margins and light cured.

**Figure 11:**
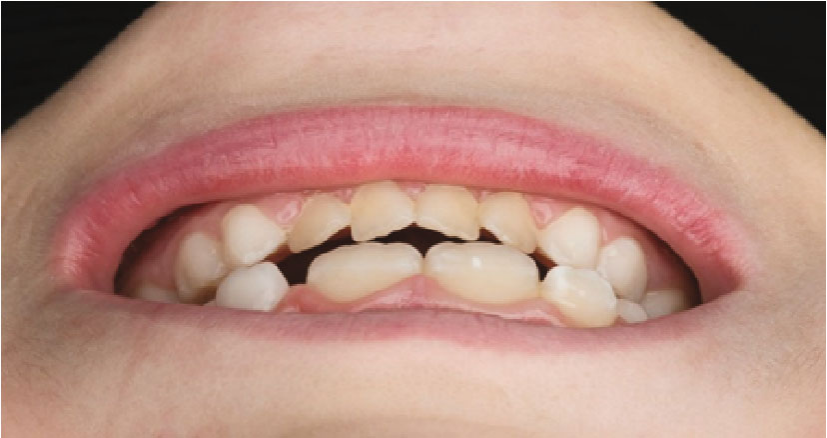
Patient's occlusion was checked for premature contacts.

**Figure 12:**
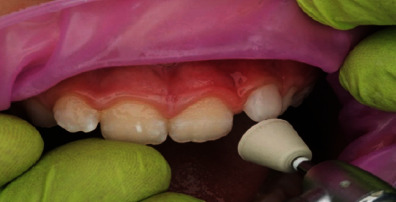
Final polishing done with polishing cups.

**Figure 13:**
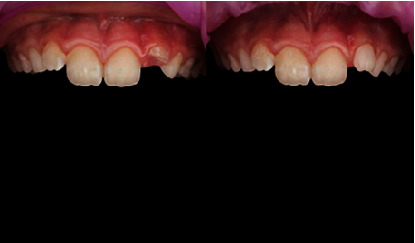
Before and after treatment showing good marginal adaptation and seal of the Edelweiss pediatric crown.

For luting, a small amount of resin composite (Shade A1, Edelweiss Dentistry Product, Graz, Austria) was placed on the inside walls of the pediatric crown's fitting surface (Figure 9). The crown was gently inserted over the prepared tooth with apical pressure to ensure a firm seat. Excess composite luting cement was removed (Figure 10), and the proper marginal seal was verified. The incisal edge and labial inclination were examined to match the opposing tooth and ensure a proper labial smile profile. The entire crown was then light cured for 20 s in all directions, including the margins, to ensure a proper marginal seal. Occlusion was checked to ensure no premature interferences during both static and dynamic occlusion (Figure 11). Final polishing was accomplished using polishing cups (Kenda polishing cups, Coltene, Altstätten, Switzerland; refer to Figure 12).  Figure 13 illustrates pre and post-treatment images of the child's tooth.

During the 1-year recall visit, the tooth was examined for signs of discoloration indicating potential vitality loss. Patient comfort and satisfaction were assessed through discussions with both the parents and the patient. No discoloration of the tooth was observed, and the gingiva appeared healthy and well maintained. The follow-up visit revealed no signs of pulp devitalization, and the patient remained pain-free. Furthermore, the pediatric crown was free from surface stains, marginal stains, or chipping. The gingiva around the crown margins exhibited a healthy and normal pinkish color. No staining or secondary caries was observed on the crown, and the margins were free of any discoloration (Figure 13). The tooth exhibited normal sensitivity to hot and cold testing, with no discoloration. Both the child and her parents found the esthetics of the crown to be acceptable and satisfactory.  Table 1 presents the list of all the procedures described in this case report.

## 3. Discussion

To begin with a pertinent digression, it is important to check for potential child abuse and ensure regulation-compliant reporting when dealing with traumatic dental injuries in children [10]. However, the child described in this case report did not have any such problems. Furthermore, the traumatic fall of the child leading to a fractured permanent lateral incisor in the maxilla was not associated with traumatic head injuries or associated neurological decrements. Thus, no referrals to other specialists were needed.

The Dental Trauma Guide and Trauma Pathfinder provides easy-to-follow recommendations and flow charts for arriving at a correct diagnosis of traumatic dental fractures and luxations of permanent teeth [10]. The diagnostic workup described in this case report was generally in line with these recommendations. However, the Dental Trauma Guide and Trauma Pathfinder recommends radiographs with periapical, occlusal, and eccentric exposures for detecting fracture lines in the root for crown fractures without pulp exposure [10].

The psychosocial decrements associated with traumatic dental injuries are well-established observations and often warrant prompt diagnosis and treatment [1, 2]. The appropriateness for the choice of full-coverage pediatric crowns as a treatment option was derived from a premise that maintaining pulp vitality and preservation of remaining tooth structure are important restorative considerations in children with traumatic injury to the tooth [7]. This premise is supported by a systematic review by Innes et al., which indicates that crowns placed on primary teeth with decay or those that have had a pulp treatment are associated with a lesser likelihood of failure or pain in the long term as compared to conventional filling [11]. Furthermore, the acceptance of crowns is high with both patients and dentists and the latter find crown-related procedures comparatively simpler even when restoring severely damaged primary molars [9]. Another study by Kaptan et al. notes that full-coverage pediatric crowns require less additional treatment and have a higher survival rate compared to conventional fillings [12].

Several materials including stainless steel, zirconia, resin composites, and hybrid glass material (Edelweiss Dentistry, Austria) can be used for fabricating full-coverage pediatric crowns. While stainless steel crowns are associated with high clinical success rates and have been recommended by the British Society of Pediatric Dentists, many dental practitioners consider stainless steel crowns unsuitable for most children as their placement entails a cumbersome restorative technique in a busy routine practice [13]. Furthermore, nickel–containing stainless-steel crowns may also carry risks of allergic reactions and hypersensitivity [14]. Over the years, the aesthetic advantages of zirconia have garnered an increased attention over stainless steel [7, 15]. However, pediatric crowns fabricated with zirconia require the removal of a higher amount of tooth structure and subgingival preparation margins of 1–2 mm to restore primary teeth [15]. These requirements can often lead to pulpal exposures because of the larger size of the pulp tissue and the much higher pulp horns in the primary tooth as compared to the permanent tooth [7]. In a comparative study, Subramanian et al. point out that zirconia crowns require more tooth reduction than stainless steel crowns and fiberglass crowns [15] Furthermore, zirconia crowns are made from rigid ceramic materials not amenable to crimping and undercuts that facilitate a free path of insertion for the zirconia crowns [7].

The consequent increase in preparation times and fitting times for zirconia crowns may increase the difficulties associated with the chairside time for a child. The rigidity of ceramic materials in zirconia crowns is especially bothersome when the crown is placed in hyperocclusion with a corresponding need for cutting or adjusting the opposing tooth into occlusion [7, 16]. Furthermore, tooth preparations that are subgingival can cause gingival bleeding and may compromise the retention of the zirconia crown [16].

Due to these limitations of stainless steel and zirconia, the use of a recently launched hybrid glass material from Edelweiss, Austria, was considered for fabricating the pediatric full crown. As per the manufacturer's specifications, the pediatric full crown fabricated using the novel hybrid glass material requires minimally invasive tooth preparation and has acceptable aesthetic properties [17]. This novel hybrid glass material is composed mainly of silica and barium glass, which imparts a natural enamel-like appearance to the crowns [17]. This hybrid glass material is manufactured through a unique process of laser sintering and vitrification of composite for improving both the compressive strength and aesthetic properties [17]. The hybrid glass material also contains a small amount of resin to enhance bonding to the tooth structure and for ease of adjustment and repair in the mouth [17]. Furthermore, the presence of fluoride and zinc oxide nanoparticles in this hybrid glass material renders useful antibacterial properties to the ensuing pediatric crowns [16]. As per the manufacturer, the hybrid glass material is devoid of Bisphenol A and, thus, has the advantages of safety in a child and is biosustainable [17]. The prefabricated pediatric crowns made from this hybrid glass material are supplied in a variety of sizes along with a sizing guide. Moreover, the shape of these prefabricated crowns closely mimics the anatomy of the deciduous tooth.

Another challenge in treating the child described in this report was the ability of the restoration to bond to the little remaining tooth structure. This challenge was addressed using a specific bond from the manufacturer, which aids in bonding the remaining tooth structure to the presilanized glass phase of the crown [17]. This claim of the manufacturer for the longevity of bonding was confirmed at 1-year follow-up, where there was no marginal staining or loosening of the restoration. In line with our finding, Novelli et al. in their article note that this type of bonding system obtained long-lasting bonds of veneers to teeth that were affected by amelogenesis imperfecta [18]. Maintaining the vitality of the pulp was another important consideration in this case. The application of the pediatric full crown fabricated using the hybrid glass material achieved optimal bonding without additional preparation of the existing fractured tooth meant preserving the remaining tooth structure. This prevented further drilling and preservation of dentine that was protecting the pulpal area. The current study demonstrates the usefulness of preformed crowns in a pediatric patient. Future studies are needed to test these prosthodontic frameworks in combination with low-noise instruments [19] and computerized devices for anesthesia [20], which may ease and enhance the experience of such frameworks in children.

## 4. Conclusion

Overall, this case report underscores the usefulness of a conservative approach for restoring an erupting permanent tooth in the mixed dentition using a pediatric full crown fabricated using a novel hybrid glass material. Improvements in function and esthetics were achieved along with preserving tooth vitality, minimal stress, and clinically noticeable child and parental satisfaction. These initial findings merit further investigation in future clinical studies.

## Figures and Tables

**Table 1:** List of procedures performed in the case.

**Step**	**Procedure**	**Details**
1	Initial assessment	• Ruling out child abuse • Assessment of ASA Physical Status Classification • Physical and neurological examination • Percussion tests to rule out luxation or root fracture • Evaluation of occlusion and bite force

2	Clinical examination	• Examination of face, lips, oral musculature • Assessment of soft tissue lesions • Palpation of jaws • Pulp sensitivity test

3	Diagnosis	• Identification of dentine fracture (Tooth 22) • Confirmation of pain and sensitivity due to exposed dentine

4	Treatment plan	• Conservative treatment approach • Selection of novel pediatric full crown made of hybrid glass material

5	Crown selection	• Selection of Shade A1 • Selection of size

6	Tooth preparation	• Minimal tooth preparation • Contouring crown margin with diamond football bur (RA 379) and Soflex discs • Maintaining supragingival crown margin

7	Crown fitting	• Roughening fitting surface of the crown with diamond football bur • Application of veneer bond and light curing

8	Tooth surface preparation	• Etching enamel with 37% phosphoric acid • Application of universal adhesive bonding • Light curing for 20 s

9	Luting of Crown	• Application of resin composite (Shade A1) inside crown • Insertion of crown with apical pressure • Removal of excess composite luting cement • Light curing for 20 s

10	Occlusion check	• Checking static and dynamic occlusion for interferences

11	Final polishing	• Polishing with Kenda polishing cups

12	Follow-up visit	• Examination for tooth discoloration and vitality • Assessment of patient comfort and satisfaction • Checking for surface stains, marginal stains, chipping, and secondary caries Verifying healthy gingiva and normal tooth sensitivity

## Data Availability

Data will be available from the corresponding author upon reasonable request.
